# Short-term pre- and post-operative stress prolongs incision-induced pain hypersensitivity without changing basal pain perception

**DOI:** 10.1186/s12990-015-0077-3

**Published:** 2015-12-02

**Authors:** Jing Cao, Po-Kai Wang, Vinod Tiwari, Lingli Liang, Brianna Marie Lutz, Kun-Ruey Shieh, Wei-Dong Zang, Andrew G. Kaufman, Alex Bekker, Xiao-Qun Gao, Yuan-Xiang Tao

**Affiliations:** Department of Anatomy, College of Basic Medicine, Zhengzhou University, Zhengzhou, 450001 Henan China; Department of Anesthesiology, New Jersey Medical School, Rutgers, The State University of New Jersey, 185 S. Orange Ave., MSB, F-548, Newark, NJ 07103 USA; Department of Anesthesiology, Buddhist Tzu Chi General Hospital, Institute of Medical Sciences, School of Medicine, Tzu Chi University, Hualien, Taiwan; Department of Anesthesiology and Critical Care Medicine, Johns Hopkins University School of Medicine, Baltimore, MD 21205 USA; Department of Physiology, School of Medicine, Institute of Medical Sciences, Tzu Chi University, Hualien, Taiwan

**Keywords:** Short-term immobilization, Short-term forced swimming, Stress, Postsurgical pain, Incision

## Abstract

**Background:**

Chronic stress has been reported to increase basal pain sensitivity and/or exacerbate existing persistent pain. However, most surgical patients have normal physiological and psychological health status such as normal pain perception before surgery although they do experience short-term stress during pre- and post-operative periods. Whether or not this short-term stress affects persistent postsurgical pain is unclear.

**Results:**

In this study, we showed that pre- or post-surgical exposure to immobilization 6 h daily for three consecutive days did not change basal responses to mechanical, thermal, or cold stimuli or peak levels of incision-induced hypersensitivity to these stimuli; however, immobilization did prolong the duration of incision-induced hypersensitivity in both male and female rats. These phenomena were also observed in post-surgical exposure to forced swimming 25 min daily for 3 consecutive days. Short-term stress induced by immobilization was demonstrated by an elevation in the level of serum corticosterone, an increase in swim immobility, and a decrease in sucrose consumption. Blocking this short-term stress via intrathecal administration of a selective glucocorticoid receptor antagonist, RU38486, or bilateral adrenalectomy significantly attenuated the prolongation of incision-induced hypersensitivity to mechanical, thermal, and cold stimuli.

**Conclusion:**

Our results indicate that short-term stress during the pre- or post-operative period delays postoperative pain recovery although it does not affect basal pain perception. Prevention of short-term stress may facilitate patients’ recovery from postoperative pain.

## Background

Persistent postsurgical pain, a pain syndrome that can develop after surgery, is a significant public health problem. Approximately 50 % of surgical patients suffer from persistent pain after surgery, of whom at least 5–10 % have severe pain [1]. The condition affects their quality of life and has important legal and medico-economic ramifications. Pharmacological management of persistent surgical pain conditions are dominated by two classes of medications: opioids and nonsteroidal anti-inflammatory drugs. Many of these painkillers have limited effectiveness or serious side effects, such as nausea/emesis, constipation, tolerance or hyperalgesia [1–4]. Understanding the factors that may cause and/or affect the development and maintenance of persistent surgical pain may provide insight into novel prevention or treatment strategies.

Although many forms of injury and stress occur spontaneously, patients know the precise timing of the elective surgical insult and ensuing pain in advance. In addition to surgical factors, psychosocial, socio-environmental, and patient-related factors appear to modulate risk of developing persistent postsurgical pain. Several psychosocial risk factors have been identified, including anxiety, depression, pain catastrophizing, and fear of surgery [4]. Self-reported sleep disturbance-induced stress before and after surgery also constitutes the strongest determinant of postsurgical pain [5].

Substantial evidence from clinical observations has demonstrated that sleep deprivation-caused chronic stress increased basal pain perception in healthy subjects [6, 7]. Some surgical patients with chronic severe physical or psychological stress self-reported increased basal pain sensitivity or exacerbated existing pathological pain before surgery [8, 9]. In preclinical studies, chronic stress induced by immobilization exacerbates mechanical allodynia after peripheral nerve injury [10]. Sleep disturbance-induced chronic stress leads to thermal hyperalgesia and an increased response to electrical stimulation in intact, experimental animals [6, 11–13]. Nevertheless, most surgical patients have normal pain perception before surgery although they have stress caused by risk factors such as depression, anxiety, fear of pain and surgery, and/or sleep disturbance for several days before and/or after surgery [4, 5]. Our recent findings suggest that pre- and post-surgical short-term sleep disturbance did not affect basal pain perception but did delay postsurgical pain recovery [14]. Whether or not short-term stress caused by other risk factors affects the recovery from postoperative pain is still elusive.

To model short-term stress caused by depression, the present study utilized two preclinical animal models of stress—immobilization stress and forced swimming stress [15–17]—to determine the optimal duration of immobilization or forced swimming that did not alter basal paw withdrawal responses to thermal, cold, and mechanical stimuli. We then determined whether the short-term immobilization or forced swimming under optimal conditions enhanced the magnitude and/or duration of thermal, cold, or mechanical hypersensitivity induced by a hind paw incision. Finally, we examined whether this short-term immobilization or forced swim produced other physiological or behavioral signs of stress.

## Results

### Time-dependent changes in basal paw withdrawal responses to mechanical, thermal, and cold stimuli after repeated immobilization stress in male rats

**Fig. 1:**
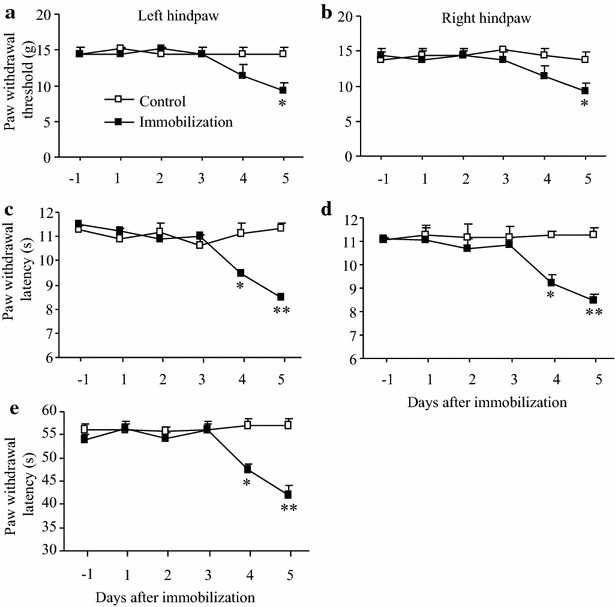
Time-dependent changes in basal paw withdrawal responses in male rats to mechanical, heat, and cold stimuli after immobilization 6 h daily for consecutive days. **a**, **b** Mechanical stimuli. **c**, **d** Heat stimuli. **e** Cold stimuli. **a**, **c**, **e** Responses of left paws. **b**, **d** Responses of right paws. Significant reductions were seen in bilateral paw withdrawal thresholds in response to mechanical stimulation on day 5 post-immobilization (**a**, **b**), in bilateral paw withdrawal latencies in response to heat stimulation on day 4 and 5 post-immobilization (**c**, **d**), and in paw withdrawal latency in response to cold stimulation on days 4 and 5 post-immobilization (**e**) in the immobilization stress group. Mean ± SEM. *P < 0.05, **P < 0.01 vs the corresponding time points in the control group. N = 5/group

We first defined the optimal number of daily repetitions of immobilization stress that did not affect basal paw withdrawal responses in an established animal model of immobilization stress. Male rats were exposed to immobilization stress for 6 h daily for 5 consecutive days. Paw withdrawal responses to mechanical, thermal, and cold stimuli were examined 1 day before immobilization stress and 2 h after immobilization stress daily for 5 days. Significant decreases in paw withdrawal thresholds in response to mechanical stimulation on both left and right hind paws were observed only on day 5 post-immobilization stress as compared to the corresponding control group (*P* < 0.05 in Fig. 1a, b), an indication of mechanical allodynia. Marked reductions in paw withdrawal latencies in response to thermal stimulation were seen on day 4 (*P* < 0.05) and 5 (*P* < 0.01) post-immobilization stress on both left and right hind paws as compared to the corresponding control group (Fig. 1c, d), an indication of thermal hypersensitivity. Similarly, significant reductions in paw withdrawal latency in response to cold stimulation were detected both on day 4 (*P* < 0.05) and 5 (*P* < 0.01) after immobilization stress in the left hind paw as compared to the corresponding control group (Fig. 1e), an indication of cold allodynia. These data showed that basal paw withdrawal responses did not change significantly during 3 consecutive days of exposure to immobilization stress. Thus, 6 h immobilization for 3 consecutive days was defined as short-term immobilization stress and was used in the following experiments.

### Effect of pre-surgical exposure to short-term immobilization stress on postsurgical pain in male rats

**Fig. 2:**
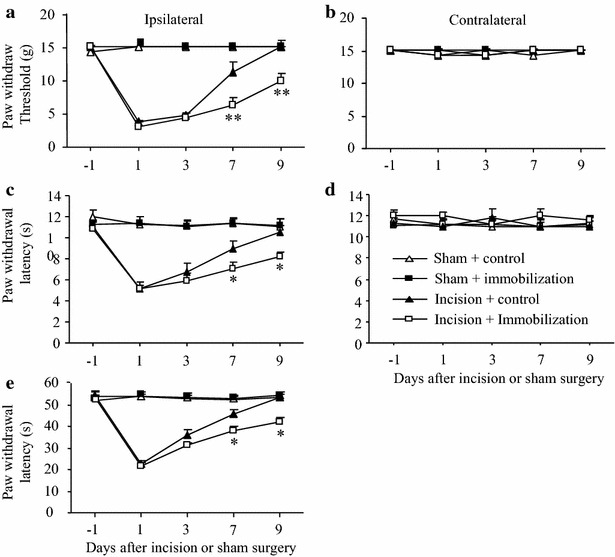
Effect of pre-surgical exposure to short-term immobilization on post-surgical pain in male rats. **a**, **b** Mechanical stimuli. **c**, **d** Heat stimuli. **e** Cold stimuli. **a**, **c**, **e** Responses of ipsilateral (incision-stressed) paws. **b**, **d** Responses of contralateral paws. Pre-surgical exposure to short-term immobilization markedly delayed recovery in the paw withdrawal threshold to mechanical stimulation (**a**) and paw withdrawal latencies to heat (**c**) and cold (**e**) stimuli on the ipsilateral side on days 7 and 9 post-surgery in the incision plus immobilization group, compared to the incision plus control group. The sham plus control and the sham plus immobilization groups showed no significant differences in paw withdrawal responses during the observation period. Mean ± SEM. *P < 0.05, **P < 0.01 vs the corresponding time points in the incision plus control group. N = 5/group

To examine whether short-term immobilization stress before surgery affected the magnitude or duration of incision-induced hypersensitivity, we carried out unilateral plantar incision on the left hind paw in male rats after they were exposed to 6 h immobilization for three consecutive days. Consistent with previous reports [18–20], the incision alone led to persistent mechanical, thermal, and cold pain hypersensitivities on the ipsilateral (but not contralateral) side of the incision plus control group (Fig. 2). Pain hypersensitivity reached a peak on day 1, lasted for 4–7 days, and had completely disappeared on day 9 post-surgery (Fig. 2a, c, e). As expected, pre-exposure to 3 days immobilization stress alone (sham plus immobilization treated rats) did not alter basal paw responses to mechanical, heat, or cold stimuli during the 9 days observation period (Fig. 2). However, pre-exposure to 3 days immobilization stress significantly delayed the recovery from surgical pain, although it did not alter peak levels of incision-induced hypersensitivity to mechanical, thermal, or cold stimuli, on the ipsilateral side in the incision plus immobilization stress group (Fig. 2a, c, e). The incision plus immobilization stress group had significantly lower paw withdrawal thresholds to mechanical stimulation on days 7 (*P* < 0.05) and 9 (*P* < 0.01) post-surgery than the incision plus control group (Fig. 2a). The incision plus immobilization stress group also had significantly shorter paw withdrawal latencies to thermal stimulation on days 7 and 9 than the incision plus control group (both *P* < 0.05; Fig. 2c). Additionally, the incision plus immobilization stress group had significantly shorter paw withdrawal latencies to cold stimulation on days 7 (*P* < 0.05) and 9 (*P* < 0.01) than the incision plus control group (Fig. 2e). As expected, the sham plus control group maintained the baseline level of paw withdrawal threshold and latencies in response to the different stimuli on both sides at all-time points. The incision group showed no effects on paw withdrawal threshold and latencies in response to the different stimuli on the contralateral side (Fig. 2).

### Effect of post-surgical exposure to short-term immobilization stress on postsurgical pain in male rats

**Fig. 3:**
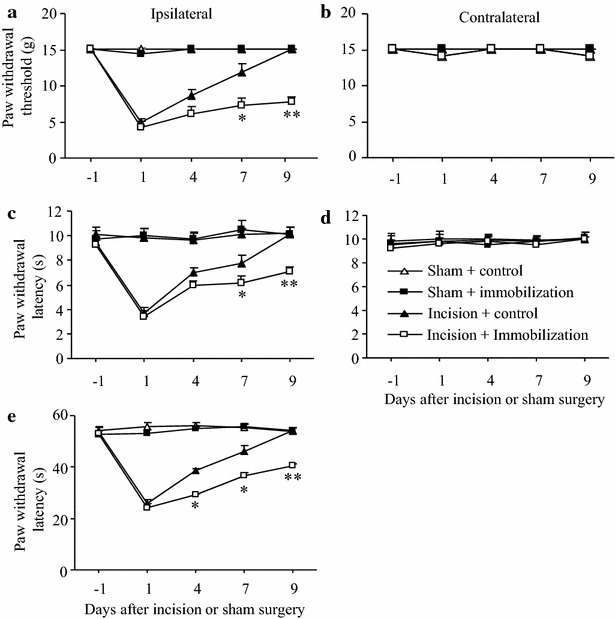
Effect of post-surgical exposure to short-term immobilization on post-surgical pain in male rats. **a**, **b** Mechanical stimuli. **c**, **d** Heat stimuli. **e** Cold stimuli. **a**, **c**, **e** Responses of ipsilateral (incision-stressed) paws. **b**, **d** Responses of contralateral paws. Post-surgical exposure to short-term immobilization markedly delayed recovery of paw withdrawal threshold to mechanical stimulation (**a**) and paw withdrawal latency to heat (**c**) on days 7 and 9 post-immobilization and paw withdrawal latency to cold (**e**) stimuli on days 4, 7, and 9 post-immobilization on the ipsilateral side in the incision plus immobilization group compared to the incision plus control group. The sham plus control and the sham plus immobilization groups showed no changes in paw withdrawal responses during the observation period. Mean ± SEM. *P < 0.05, **P < 0.01 vs the corresponding time points in the incision plus control group. N = 5/group

We next examined whether short-term immobilization stress after surgery affected the magnitude or duration of incision-induced hypersensitivity in male rats. One hour after unilateral incision on the left hind paw, male rats were exposed to 6 h immobilization stress daily for three consecutive days. Immobilization stress markedly delayed the recovery of surgical pain, although it did not alter peak levels of incision-induced hypersensitivity to mechanical, thermal, or cold stimuli, on the ipsilateral side in the incision plus immobilization stress group (Fig. 3a, c, e). The incision plus immobilization stress group had significantly lower paw withdrawal thresholds to mechanical stimulation on days 7 (*P* < 0.05) and 9 (*P* < 0.01) post-surgery than the incision plus control group (Fig. 3a). In addition, the incision plus immobilization group had shorter paw withdrawal latency to thermal stimulation on days 7 (*P* < 0.05) and 9 (*P* < 0.01) post-surgery than the corresponding incision plus control group (Fig. 3c). Likewise, the incision plus immobilization group had significantly shorter paw withdrawal latency to cold stimulation on days 4, 7 (both *P* < 0.05), and 9 (*P* < 0.01) post-surgery than the incision plus control group (Fig. 3e). As expected, basal behavioral responses were not altered on the contralateral side in all groups and on the ipsilateral sides in the sham plus control group and the sham plus immobilization group during the observation period (Fig. 3).

### Effect of post-surgical exposure to short-term immobilization stress on postsurgical pain in female rats

**Fig. 4:**
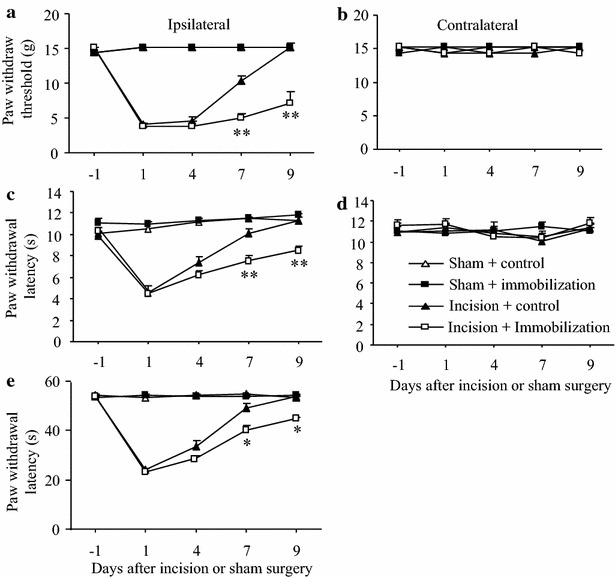
Effect of post-surgical exposure to short-term immobilization on post-surgical pain in female rats. **a**, **b** Mechanical stimuli. **c**, **d** Heat stimuli. **e** Cold stimuli. **a**, **c**, **e** Responses of ipsilateral (incision-stressed) paws. **b**, **d** Responses of contralateral paws. Post-surgical exposure to short-term immobilization markedly delayed recovery of paw withdrawal threshold to mechanical stimulation (**a**) and paw withdrawal latencies to heat (**c**) and cold (**e**) stimuli on days 7 and 9 post-immobilization on the ipsilateral side in the incision plus immobilization group, compared to the incision plus control group. No changes in paw withdrawal responses were seen during the observation period in the sham plus control and sham plus immobilization groups. Mean ± SEM. *P < 0.05, **P < 0.01 vs the corresponding time points in the incision plus control group. N = 5/group

Gender-related differences in pain and stress have been described in experimental settings and clinical observations [21, 22]. Thus, we examined whether short-term immobilization stress after surgery has stronger effects on postoperative pain in female rats than in male rats. Female rats were exposed to incision and subsequently 3 days immobilization stress in the same manner as male rats. The magnitude and duration of incision-induced pain hypersensitivity on the ipsilateral side in the female rats was similar to those in male rats (Fig. 3a, c, e vs Fig. 4a, c, e). Similar to the responses of male rats, the female incision plus immobilization stress group had a significantly slower recovery from surgical pain and an unchanged magnitude of incision-induced hypersensitivity to mechanical, thermal, or cold stimuli, on the ipsilateral side when compared to the incision plus control group (Fig. 4a, c, e). The female incision plus immobilization stress group had significantly lower mean paw withdrawal threshold to mechanical stimulation on days 7 and 9 post immobilization than the incision plus control group (both *P* < 0.01; Fig. 4a). Similarly, the female incision plus immobilization stress group had significantly shorter mean paw withdrawal latency to thermal stimulation on days 7 (*P* < 0.05) and 9 (*P* < 0.01) post immobilization (Fig. 4c) and shorter mean paw withdrawal latency to cold stimulation on days 7 and 9 post immobilization (both *P* < 0.05; Fig. 4e) than the female incision plus control group. As expected, basal behavioral responses were not changed on the contralateral side of all groups and the ipsilateral side of the sham plus control group and the sham plus immobilization group during the observation period in female rats (Fig. 4).

### Effect of post-surgical exposure to short-term forced swimming stress on postsurgical pain in male rats

**Fig. 5:**
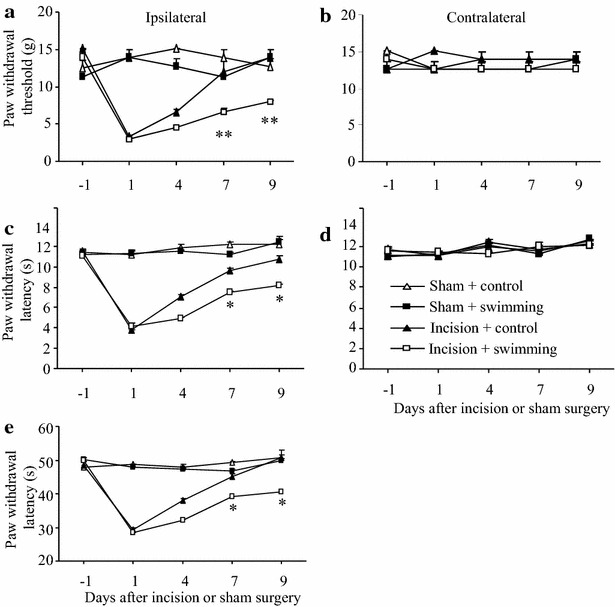
Effect of post-surgical exposure to short-term forced swimming on post-surgical pain in male rats. **a**, **b** Mechanical stimuli. **c**, **d** Heat stimuli. **e** Cold stimuli. **a**, **c**, **e** Responses of ipsilateral (incision-stressed) paws. **b**, **d** Responses of contralateral paws. Post-surgical exposure to short-term forced swimming markedly delayed recovery in paw withdrawal threshold to mechanical stimulation (**a**) and paw withdrawal latencies to heat (**c**) and cold (**e**) stimuli on days 7 and 9 post-forced swimming on the ipsilateral side in the incision plus forced swimming group compared to the incision plus control group. No changes in paw withdrawal responses were seen during the observation period in the sham plus control and the sham plus forced swimming groups. Mean ± SEM. *P < 0.05, **P < 0.01 vs the corresponding time points in the incision plus control group. N = 5/group

Our pilot work showed that basal paw withdrawal responses did not change markedly during three consecutive days of exposure to forced swimming stress (20 min daily; data not shown). Thus, we defined 20-min forced swimming for 3 consecutive days as short-term forced swimming stress. To further explore the role of short-term stress on recovery from postsurgical pain, we examined whether short-term forced swimming stress after surgery affected postoperative pain in male rats. One hour after unilateral plantar incision on the left hind paw, male rats were exposed to the forced swimming stress (20 min) daily for three consecutive days. Similar to the effects of the 3 days immobilization stress described above, post-exposure to forced swimming stress daily for 3 days also markedly delayed the recovery from surgical pain, although it did not alter the magnitude of incision-induced hypersensitivity to mechanical, thermal, or cold stimuli, on the ipsilateral side in the incision plus forced swimming stress group (Fig. 5a, c, e) compared to the recovery in the incision plus control group. The incision plus forced swimming stress group showed lower mean paw withdrawal thresholds to mechanical stimulation on days 7 and 9 post surgery than the incision plus control group (both *P* < 0.01; Fig. 5a). In addition, the incision plus forced swimming stress group showed shorter paw withdrawal latencies to thermal stimulation on days 7 and 9 post surgery (both *P* < 0.01; Fig. 5c) and shorter paw withdrawal latencies to cold stimulation on days 7 and 9 post-surgery (both *P* < 0.01; Fig. 5e). As expected, no significant changes were detected in paw withdrawal threshold and latencies on the contralateral side in all groups during the 9 days. The ipsilateral sides of the sham plus control and the sham plus swimming stress group also maintained the baseline levels of the paw withdrawal thresholds and latencies during the observation period (Fig. 5).

### Existence of stress in male rats after post-surgical exposure to 3-day immobilization stress

**Fig. 6:**
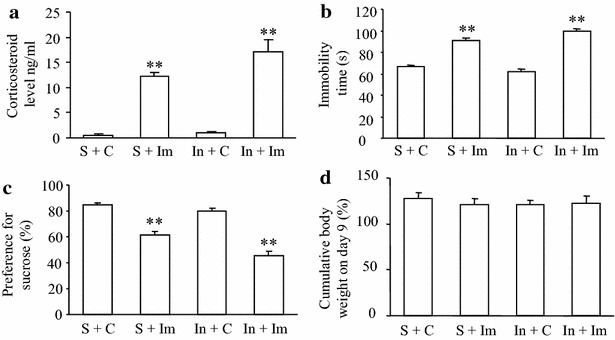
Existence of stress after short-term immobilization. **a** Corticosterone serum levels. **b** Forced swim test. **c** Sucrose consumption. **d** Body weight. Post-surgical exposure to short-term immobilization significantly elevated the level of corticosterone in serum (**a**), increased immobility time in a forced swim test (**b**), and decreased sucrose consumption in a sucrose preference test (**c**) in both the sham incision plus immobilization (S + Im) group and the incision plus immobilization (In + Im) group compared to the sham plus control (S + C) group. These changes were not observed in the incision plus control group (**a**–**c**). No significant differences in changes in body weight between before surgery and on day 9 post-surgery were seen among the four groups (**d**). Mean ± SEM. **P < 0.01 vs the corresponding sham plus control group. N = 5/group. *C* control, *Im* 3d immobilization, *In* incision, *S* sham incision

To identify the existence of the stress under our optimized conditions, we also examined post-surgical male rats used for behavioral studies described above in the following 3 tests. We first measured the level of corticosterone (CORT) in serum to determine if their stressed behavior correlated with CORT levels. The blood was collected immediately after post-surgical exposure to 6 h immobilization stress daily for three consecutive days. The levels of serum CORT in the sham plus immobilization stress group and the incision plus immobilization stress group were significantly higher than those in the sham plus control group (both *P* < 0.01, Fig. 6a). No significant difference was observed in serum CORT levels between the incision plus control group (*P* > 0.05) and the sham plus control group (Fig. 6a). We further carried out a swimming immobilization test, in which rats under stress display a longer duration of immobility [23], 1 h after blood withdrawal. The rats in the sham plus immobilization stress group and in the incision plus immobilization stress group spent significantly more time immobile than rats in the sham plus control group (*P* < 0.01; Fig. 6b). The immobility duration was similar between the incision plus control group and the sham plus control group (Fig. 6b). Finally, we examined these animals in the sucrose preference test, in which rats under stress display reduced sucrose preference [23]. The rats in the sham plus immobilization stress group and in the incision plus immobilization stress group showed significantly less sucrose consumption than those in the sham plus control group (*P* < 0.01, Fig. 6c). No obvious difference in sucrose consumption was observed between the incision plus control group and the sham plus control group (Fig. 6c). The effect of 3 days immobilization stress on body weight was not significant, as the body weights were similar among all four groups on day 9 post-incision or -sham surgery (Fig. 6d).

### Effect of intrathecal RU38486 on exacerbated post-surgical pain after short-term immobilization stress

**Fig. 7:**
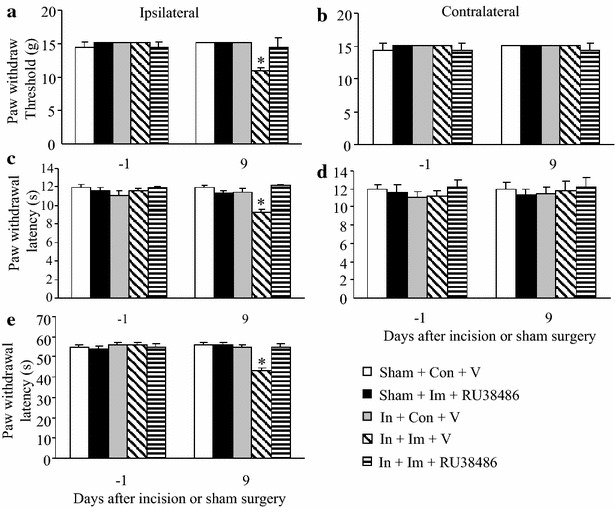
Effect of intrathecal pre-administration of RU38486 on exacerbated post-surgical pain induced by short-term immobilization stress. **a**, **b** Mechanical stimuli. **c**, **d** Heat stimuli. **e** Cold stimuli. **a**, **c**, **e** Responses of ipsilateral paws. **b**, **d** Responses of contralateral paws. The incision plus immobilization plus intrathecal vehicle (In + Im + V) group had significantly lower paw withdrawal threshold to mechanical stimulation (**a**) and shorter paw withdrawal latencies to heat (**c**) and cold (**e**) stimuli on the ipsilateral side on day 9 post-incision than the incision plus control plus vehicle group. In contrast, RU38486 (2 μg/10 μl) 1 h before each 6 h of immobilization for 3 days completely abolished these reductions (**a**, **c**, **e**). RU38486 did not alter basal paw withdrawal responses to mechanical, heat, and cold stimuli on the contralateral side of the incision plus immobilization group (**b**, **d**) and on either ipsilateral (**a**, **c**, **e**) or contralateral (**b**, **d**) side of the sham plus immobilization group. Intrathecal vehicle (V) did not affect basal paw withdrawal responses on either side of any group. Mean ± SEM. **P < 0.01 vs the corresponding sham plus control group with intrathecal vehicle. N = 5/group. *In* incision, *Im* 3d immobilization, *V* vehicle

CORT produces its physiological and behavioral effects by binding to and activating glucocorticoid receptors [24]. To determine whether increased CORT induced by immobilization under our optimized conditions contributed to the observed prolongation of incision-induced hypersensitivity, we intrathecally pre-administered the control vehicle or a selective glucocorticoid receptor antagonist RU38486 1 h before each session of 6 h immobilization stress daily for 3 days. In agreement with our aforementioned results, the incision-immobilization stress-vehicle group showed the exacerbated paw withdrawal responses to mechanical (Fig. 7a), thermal (Fig. 7c), and cold (Fig. 7e) stimuli on the ipsilateral side on day 9 post-incision compared to the corresponding the incision-control-vehicle group (all *P* < 0.05). Intrathecal pre-administration of RU38486 in the incision—immobilization stress group completely abolished these exacerbated paw withdrawal responses on the ipsilateral side on day 9 (Fig. 7a, c, e). RU38486 administration at the dose used did not alter basal paw withdrawal responses to mechanical and thermal stimuli on the contralateral side of the incision plus immobilization stress group and to mechanical, thermal and cold stimuli on either ipsilateral or contralateral side of the sham plus immobilization stress group (Fig. 7).

### Effect of bilateral ADX on exacerbated post-surgical pain after short-term immobilization stress

**Fig. 8:**
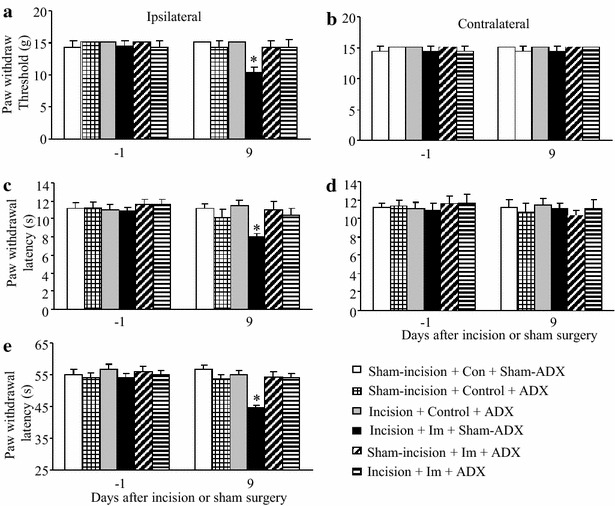
Effect of bilateral adrenalectomy (ADX) on exacerbated post-surgical pain induced by short-term immobilization stress. **a**, **b** Mechanical stimuli. **c**, **d** Heat stimuli. **e** Cold stimuli. **a**, **c**, **e** Responses of ipsilateral paws. **b**, **d** Responses of contralateral paws. In the incision plus immobilization group, sham surgery of ADX (sham-ADX) before immobilization showed marked reductions in paw withdrawal threshold to mechanical stimulation (**a**) and paw withdrawal latencies to heat (**c**) and cold (**e**) stimuli on the ipsilateral side on day 9 post-incision. Bilateral ADX before immobilization entirely reversed these reductions (**a**, **c**, **e**). ADX and sham ADX showed similar paw withdrawal responses to mechanical, heat, and cold stimuli on the contralateral sides of all groups (**b**, **d**). ADX and sham ADX did not affect the responses of the ipsilateral paws to the stimuli (**a**, **c**, **e**) of the sham-incision plus control group, the incision plus control group, and the sham-incision plus immobilization group. Mean ± SEM. **P < 0.01 vs the corresponding sham-incision plus control group with sham-ADX. N = 5/group. *Im* immobilization

Serum CORT originates mainly from adrenal glands. To further substantiate the role of CORT and RU38486′s pharmacological effect observed above in the exacerbated postsurgical pain, we carried out bilateral ADX to eliminate the production of serum CORT. Rats were given supplemented drinking water to maintain basal CORT levels after ADX surgery. The incision—immobilization stress -sham ADX surgery group showed the exacerbated paw withdrawal responses to mechanical, thermal, and cold stimuli on the ipsilateral side on day 9 post-incision (all *P* < 0.05; Fig. 8a, c, e, respectively). Bilateral ADX entirely reversed these exacerbated responses in the incision plus immobilization stress group (Fig. 8a, c, e). Bilateral ADX did not alter basal paw withdrawal responses to mechanical and thermal stimuli of the contralateral paws in all groups (Fig. 8b, d) nor those on the ipsilateral of the remaining treatment groups (Fig. 8).

## Discussion

Surgery-induced persistent pain is a common clinical symptom. Identifying risk factors that exacerbate post-surgical pain during the perioperative period may help us to predict which patients may experience delayed recovery and offer an opportunity to manage post-surgical pain effectively. Chronic stress increased basal pain perception and worsen existing pathological pain [8, 9]. However, most surgical patients have normal physiological, psychological health status and normal pain perception before surgery although they have to some extent stress during pre- and/or post-operative period. Whether this short-term stress affects persistent post-surgical pain is still elusive. We recently reported that pre- and post-surgical short-term sleep disturbance did not affect basal pain perception but did delay postsurgical pain recovery [14]. Here, we report that pre- or post-surgical exposure to short-term stress prolongs the duration of incision-induced mechanical, thermal, or cold hypersensitivities even if it does not alter basal nociception. Our findings suggest that prevention of short-term stress during pre- and post-operative period may help the patients recover from postoperative pain.

Immobilization and forced swimming are established stressors in rodents as defined by their effectiveness to activate the hypothalamic–pituitary–adrenal (HPA) axis and produce behavioral signs of depression [15–17]. We established two regimens of immobilization and forced swimming that did not alter basal responses to mechanical, thermal, and cold stimuli in naïve rats. These exposures to immobilization and forced swimming may model clinical conditions in most patients that have normal pain perception before surgery, although they may experience transient HPA axis activation in association with short-term fear of pain, anxiety, depression, and pain catastrophizing during pre- and post-operative periods. Evidence of stress in our current models is demonstrated by increased immobility time in a forced swim test, decreased preference for sucrose, and elevated serum CORT levels. Interestingly, short-term immobilization or forced swimming stress either before or after surgery prolonged post-surgical pain, although it did not alter the magnitude of incision-induced hypersensitivity to mechanical, thermal, or cold stimuli. Blocking spinal glucocorticoid receptor or removing the adrenal glands (that are the primary source of CORT) completely abolished the prolongation of incision-induced hypersensitivity after immobilization stress. Given that glucocorticoid receptor is expressed predominantly in the neurons of spinal cord and dorsal root ganglion [25–27], these results indicate that CORT-triggered activation of glucocorticoid receptors in spinal cord and dorsal root ganglion (rather than in brain) neurons may mediate this prolongation. Intrathecal exogenous CORT would be expected to delay the recovery of surgical pain. This expectation is strongly supported by a previous study showing that systemic exogenous administration of CORT reduced the pain threshold in the ipsilateral hind paw after peripheral nerve injury [10]. It should be noted that the ADX cannot rule out the involvement of other hormones (e.g., catecholamines) in short-term stress-induced prolongation of post-surgical pain as both adrenal cortex and medulla are removed. Unexpectedly, both male and female rats exhibited similar magnitudes and durations of postsurgical pain in either presence or absence of immobilization stress in our observations. Sex differences in response to pain and stress have been reported [21, 22]. No sex difference in our observation may be related to the short-term immobilization stress and no gender distinct in incisional pain [28].

The distinct mechanisms affected by short-term stress that do not alter basal pain perception but do prolong incision-induced hypersensitivity are unknown, but may involve one or more of the following signaling molecules and processes. Nitric oxide synthase-containing magnocellular neurons of the rat hypothalamus, which contribute to the descending projection to the spinal cord, express Fos following stress [29]. Repeated stress reduces ADP hydrolysis [30], increases 5′-nucleotidase activity [30], and produces apoptosis [31] in the spinal cord. More interestingly, immobilization stress not only exacerbates nerve injury-induced mechanical allodynia but also enhances nerve injury-induced expression of extracellular signal-regulated kinase phosphorylation in the superficial dorsal horn of spinal cord [10]. Blocking spinal cord NMDA receptor prevents stress-induced exacerbation of allodynia after spared nerve injury [10]. It was reported that spinal cord NMDA receptor is not involved in central mechanisms underlying postoperative pain in non-stressed animals [32, 33]. Whether the role of NMDA receptors and the signaling components described above participate in the short-term stress-induced prolongation of incisional pain in the present study remains to be further defined. Not without saying, other potential mechanisms cannot be ruled out.

## Conclusions

In conclusion, the present study showed that short-term stress does not affect basal nociception but does prolong post-surgical hypersensitivity to mechanical, cold, and thermal stimuli applied to the incision site. Although the detailed mechanisms underlying this effects are still unclear, our findings indicate that prevention of short-term CORT signaling during pre and post-operative periods may facilitate recovery from postoperative pain for patients.

## Methods

### Animal preparation

All male and female Sprague–Dawley rats weighing 200–300 g were obtained from Charles River Laboratories (Wilmington, MA, USA) and were housed in an animal facility that was kept in a standard 12-h light/dark cycle, with standard laboratory water and food pellets available ad libitum. Rat experiments were conducted with the approval of the Animal Care and Use Committee at New Jersey Medical School and were consistent with the ethical guidelines of the US National Institutes of Health and the International Association for the Study of Pain. All efforts were made to minimize animal suffering and to reduce the number of animals used. To minimize intra- and inter-individual variability of behavioral outcome measures, animals were habituated 2 h daily for 2 days before behavioral testing was performed. The experimenters could not be blinded to incision, but were blinded to drug/stress treatments or adrenalectomy during behavioral testing.

### Incisional pain model

The incisional surgery was carried out as described [18] with the following minor modifications. After animals were anesthetized with 2 % isoflurane delivered via a nose cone, the plantar aspect of the left hindpaw was prepared sterilely (10 % povidone-iodine solution) and incised longitudinally (1 cm; number 11 blade through the skin and fascia) from 0.5 cm from the proximal edge of the heel toward the toes. The plantaris muscle was elevated and incised longitudinally. After hemostasis with gentle pressure, the skin was sutured with 5–0 nylon. After surgery, the animals were allowed to recover in their cages. Typically, the wound healed well within 5–6 days.

### Immobilization stress

Immobilization stress, a strong non-invasive physical stressor with psychological components, was carried out for 6 h (between 8 am and 2 pm) without food and water daily in the cage on 3–5 consecutive days as described [15, 16]. Briefly, the rats were immobilized with metal mesh restrainers secured at the head and tail ends with clips to restrict the motion of the head and body. Control rats were housed in their usual cages under normal conditions.

### Forced swimming stress

Forced swimming for 20 min in the morning of 3 consecutive days was carried out individually in a vertical Plexiglas cylinder (diameter 30 cm, height 50 cm) filled with 24 ± 1 °C water at a 25 cm depth as described [27]. Briefly, rats, unable to touch the bottom with their hind paws, were considered to be swimming when they moved around the container with all four paws. Rats from the sham group were subjected to a sham swimming session by allowing them to wade in the cylinder that contained only 2 ± 4 cm of water at 24 ± 1 °C. The rats were dried before being returned to their home cages. The water was changed after each session.

### Intrathecal catheter implantation and drug administration

Intrathecal catheters (polyethylene 10) were implanted into the subarachnoid space between L4 and L5 vertebrae and advanced 2–2.5 cm into the lumbar enlargement of the spinal cord as described [34] before drug administration. The residual catheter was tunneled under skin to the neck area and the outer part of the catheter was exposed, carefully plugged, and fixed onto the skin. Animals received 2000 U of penicillin to prevent infection. The rats were allowed to recover for 5–7 days. None of the animals exhibited postoperative neurological deficits (e.g., paralysis) or poor grooming habits after catheter insertion surgery.

Mifepristone (RU38486, 2 μg, Sigma–Aldrich, St. Louis, MO, USA) dissolved in a 10 % ethanol solution (vehicle) or vehicle alone (10 % ethanol) was administered intrathecally in a 10 μl volume followed by a 10 μl saline flush 1 h prior to immobilization stress daily for 3 days. The dosage of RU38486 used was based on a previous report [25].

### Bilateral adrenalectomy

Bilateral adrenalectomy (ADX) was performed through two dorsolateral midflank skin and muscular incisions as described [35]. The ADX rats were supplemented with 25 μg/ml CORT (Sigma–Aldrich, St. Louis, MO, USA) in the drinking saline to presumably maintain basal levels of CORT and its circadian rhythmicity. Fresh solution was prepared every 2 days. The sham-ADX (Sham) rats underwent the same procedure except for the removal of the adrenal glands. The sham rats received drinking saline (without CORT) instead of drinking water after surgery. All rats were allowed to recover for 1 week before the experiments.

### Behavioral analysis

All behavioral tests were carried out in the afternoons.

Paw withdrawal thresholds in response to mechanical stimuli were measured with the up–down testing paradigm described previously [34, 36]. Briefly, rats were placed in Plexiglas chambers on an elevated mesh screen. Von Frey filaments in log increments of force (0.407, 0.692, 1.202, 2.041, 3.63, 5.495, 8.511, 15.14 g) were applied to the plantar surface of the rats’ left and right hind paws. The 2.041-g stimulus was applied first. If a positive response occurred, the next smaller von Frey hair was used; if a negative response was observed, the next larger von Frey hair was used. The test was terminated when (1) a negative response was obtained with the 15.14-g hair or (2) three stimuli were applied after the first positive response. Paw withdrawal threshold was determined by converting the pattern of positive and negative responses to the von Frey filament stimulation to a 50 % threshold value with a formula provided by Dixon [37].

Paw withdrawal latencies to noxious heat were measured with a Model 336 Analgesic Meter (IITC Inc./Life Science Instruments, Woodland Hills, CA, USA) as described previously [34, 37]. Briefly, a beam of light that provided radiant heat was aimed at the middle of the plantar surface of each hind paw. When the animal lifted its foot, the light beam turned off. Paw withdrawal latency was defined as the number of seconds between the start of the light beam and the foot lift; an allowed maximum of 20 s avoided tissue damage to the hind paw. Each trial was repeated five times at 5-min intervals for each side.

Paw withdrawal latencies to noxious cold (0 °C) were measured with a cold plate as described [34, 36]. Briefly, the paw withdrawal latency was defined as the number of seconds between placement of the hind paw on the 0 °C plate and the rapid withdrawal of the hind paw, with or without paw licking and biting. An allowed maximum of 60 s avoided paw tissue damage. Each trial was repeated three times at 10-min intervals for the paw on the ipsilateral side.

Swimming immobilization test was carried out as described above in the forced swimming stress. Briefly, swimming immobilization for each rat was defined as the minimal movement necessary to stay afloat. The duration of immobilization was recorded during the 5-min testing period.

### Sucrose preference test

Sucrose preference test was performed as a two-bottle choice paradigm (water or 1 % sucrose) described previously [23]. After rats were trained for 2 days with the two drinking bottles, the individually housed rats had access to randomly placed (left vs right side of the cage) bottles from one trial to another. After stress, each rat’s consumption of water and sucrose during a 24 h period was determined using a standard weight scale. Sucrose preference was calculated using the following format: sucrose intake (g)/[sucrose intake (g) + water intake (g)].

### Blood collection and corticosterone levels

To measure the level of CORT in the blood after the stress test without interference from the stress of animal handling, the blood (500 μl) was collected via a retro-orbital bleeding procedure as described [38, 39] in lightly anesthetized rats (isoflurane) within 3 min as the CORT levels were elevated after 3 min of animal handling. After the samples were centrifuged for 10 min at 4000 rpm at 4 °C, the supernatant plasma was collected, aliquoted, and stored at −80 °C. The CORT levels were detected using a corticosterone ELISA kit (Enzo Life Sciences, Inc. Farmingdale, NY, USA).

### Measurement of body weight

Rats were weighed before stress or incision and on day 9 after incision.

### Statistical analysis

Results were collected by scientists blinded to the treatments and reported as mean ± SEM. The data were analyzed with a one-way or two-way ANOVA. When an ANOVA test showed a significant difference, pairwise comparisons between means were tested by the post hoc Tukey method (SigmaStat, San Jose, CA, USA). Significance was set at *p* < 0.05.

## Abbreviations

ADX: adrenalectomy
C: control
CORT: corticosterone
HPA: hypothalamic–pituitary–adrenal
In: incision
Im: immobilization stress (3 consecutive days of 6 h of immobilization)
NMDA: *N*-Methyl-d-aspartate
V: vehicle
